# Acquisition of resistance to carbapenem and macrolide-mediated quorum sensing inhibition by *Pseudomonas aeruginosa* via ICE_Tn*4371*_*6385*

**DOI:** 10.1038/s42003-018-0064-0

**Published:** 2018-05-31

**Authors:** Yichen Ding, Jeanette W. P. Teo, Daniela I. Drautz-Moses, Stephan C. Schuster, Michael Givskov, Liang Yang

**Affiliations:** 1 Interdisciplinary Graduate School (IGS), Nanyang Technological University, 50 Nanyang Avenue, Singapore, 639798 Singapore; 2 Singapore Centre for Environmental Life Sciences Engineering (SCELSE), Nanyang Technological University, 60 Nanyang Drive, Singapore, 637551 Singapore; 3 School of Biological Sciences, Nanyang Technological University, 60 Nanyang Drive, Singapore, 637551 Singapore; 4 Microbiology Unit, Department of Laboratory Medicine, National University Hospital, Singapore, 119074 Singapore; 5 Department of Immunology and Microbiology, Costerton Biofilm Center, University of Copenhagen, 2200 København N Copenhagen, Denmark

## Abstract

*Pseudomonas aeruginosa* can cause life-threatening infections in immunocompromised patients. The first-line agents to treat *P. aeruginosa* infections are carbapenems. However, the emergence of carbapenem-resistant *P. aeruginosa* strains greatly compromised the effectiveness of carbapenem treatment, which makes the surveillance on their spreading and transmission important. Here we characterized the full-length genomes of two carbapenem-resistant *P. aeruginosa* clinical isolates that are capable of producing New Delhi metallo-β-lactamase-1 (NDM-1). We show that *bla*_NDM-1_ is carried by a novel integrative and conjugative element (ICE) ICE_Tn*4371*_*6385*, which also carries the macrolide resistance gene *msr(E)* and the florfenicol resistance gene *floR*. By exogenously expressing *msr(E)* in *P. aeruginosa* laboratory strains, we show that Msr(E) can abolish azithromycin-mediated quorum sensing inhibition in vitro and anti-Pseudomonas effect in vivo. We conclude that ICEs are important in transmitting carbapenem resistance, and that anti-virulence treatment of *P. aeruginosa* infections using sub-inhibitory concentrations of macrolides can be challenged by horizontal gene transfer.

## Abstract (web summary)

Yichen Ding et al. identify a novel integrative and conjugative element that confers *Pseudomonas aeruginosa* with resistance to carbapenem, the last-resort drug for susceptable Gram-negative bacterial infections. This study also shows how antivirulence treatment for *P. aeruginosa*infections can be challenged by horizontal gene transfer.

## Introduction

P*seudomonas aeruginosa* is an opportunistic pathogen which can cause life-threatening infections in immunocompromised patients^1^. It is also responsible for chronic wound infections, ventilator-associated pneumonia and chronic airway infections in cystic fibrosis and chronic obstructive pulmonary disease patients^2–6^. These infections are usually very difficult to eradicate and associated with high mortality rates^3,7^.

Carbapenems such as imipenem and meropenem are the first-line agents for treating *P. aeruginosa* infections and the last-resort drugs for severe infections caused by other susceptible Gram-negative pathogens^8^. However, the clinical efficacy of carbapenems has been greatly compromised by the spreading of the carbapenemase New Delhi metallo-β-lactamase-1 (NDM-1), which is usually encoded and transmitted by broad-host self-conjugative plasmids in Enterobacteriaceae spp. and *Acinetobacter baumannii*^9^. It was later reported that *bla*_NDM-1_ was identified in *P. aeruginosa* isolated from North America, Europe and Asia, suggesting their prevalence worldwide^10–16^. The NDM-1-producing *P. aeruginosa* strains usually have high resistance to carbapenems, as well as multidrug resistance to other antibiotics, which leaves only a handful of antibiotics effective to treat their infections in clinical practice^10–16^. Therefore, it is important to develop novel treatment strategies for *P. aeruginosa* infections.

One strategy to treat *P. aeruginosa* infections is by inhibiting the production of virulence factors, which are essential for its pathogenesis. For instance, the type III secretion system and the secreted products such as elastase and exotoxin A were shown to play important roles during the colonization of *P. aeruginosa* in human airways^17,18^. The secreted detergent rhamnolipid causes rapid necrosis of the host immune cells and protects bacterial cells from immune attack to facilitate the establishment of infections^19^. In *P. aeruginosa*, the expression of many virulence factors is under tight control of its quorum sensing systems, which can be potential targets for the design of anti-virulence drugs^20,21^. Although several anti-quorum sensing compounds have been identified in the past, none of them has so far entered clinical trial^21–23^. On the other hand, the macrolide antibiotics such as erythromycin, azithromycin (AZM) and clarithromycin were shown to have a promising anti-quorum sensing activity, which makes them ideal anti-virulence drugs for treating *P. aeruginosa* infections^21^. It was reported that macrolides can repress the synthesis of quorum sensing signaling molecules by interfering with the signaling pathways of RsmZ and RsmY through yet-to-be identified targets, thereby inhibiting the production of quorum sensing-regulated virulence products such as rhamnolipids and elastase in *P. aeruginosa*^24^. The use of macrolides as quorum sensing inhibitors has expanded our antimicrobial arsenal against *P. aeruginosa* infections until new and more efficient anti-virulence drugs become clinically available.

Although NDM-1-producing *P. aeruginosa* strains have been reported worldwide^10–16^, their complete genome information is still lacking. In this study, we sequenced the complete genomes of two NDM-producing *P. aeruginosa* strains. Comparative genomic analysis showed that *bla*_NDM-1_ is carried by a novel Tn*4371* family integrative and conjugative element (ICE), which is a class of mobile genetic element present in genomes of a broad range of β- and γ-proteobacteria^25^. In addition to *bla*_NDM-1_, this element also carries the macrolide resistance gene *msr(E)* and the florfenicol resistance gene *floR*. We show that the expression of *msr(E)* in *P. aeruginosa* can abolish AZM-mediated quorum sensing inhibition in vitro and anti-Pseudomonas effect of AZM in vivo.

## Results

### Identification of a novel NDM-1-producing PASGNDM group

Eleven multidrug-resistant *P. aeruginosa* strains were previously isolated from patients in a hospital in Singapore. These strains are resistant to carbapenems, cephalosporins, aminoglycosides and fluoroquinolones, whereas they remained sensitive to polymyxin B (Supplementary Table 1). To characterize the resistance mechanisms and the epidemiological link among these isolates, we sequenced their draft genomes on an Illumina MiSeq platform. We found that the 11 genomes are highly similar to each other as shown by multiple genome alignment (Supplementary Fig. 1) and carried the same sets of antibiotic resistance genes, suggesting that the 11 NDM-1-producing *P. aeruginosa* strains isolated in this outbreak belong to the same phylogenetic group. We therefore named this closely related *P. aeruginosa* group PASGNDM.

**Fig. 1:**
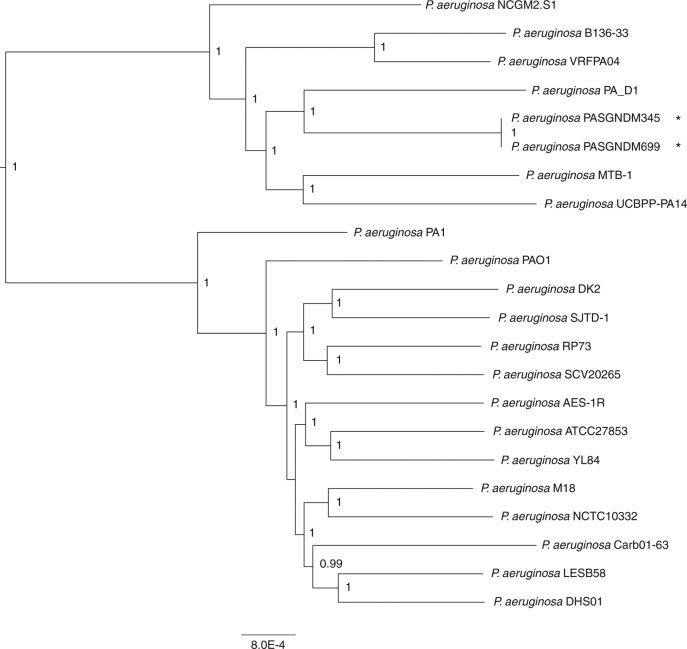
Phylogenetic tree of PASGNDM345 and PASGNDM699 with 21 *P. aeruginosa* genomes. PASGNDM345 and PASGNDM699 genomes (indicated with *) were compared with 21 other *P. aeruginosa* genomes. Phylogenetic tree was constructed based on detected variant sites of core-genome alignment using the approximate maximum likelihood algorithm, with clade confidence estimated with SH-like support values. The scale bar shows substitutions per core-genome site. Accession numbers of genomes used to construct the phylogenetic tree are listed in Supplementary Table 2

To better understand the detailed features of the PASGNDM genomes and the transmission of *bla*_NDM-1_, we further sequenced PASGNDM345 and PASGNDM699 genomes on a Pacific Biosciences RS II platform. The PacBio sequencing reads achieved 163- and 161-fold coverages for PASGNDM345 and PASGNDM699 genomes respectively and were successfully assembled into two full-length genomes. Construction of phylogenetic tree with PASGNDM345 and PASGNDM699 genomes and other 21 *P. aeruginosa* full-length genomes showed that the two PASGNDM strains formed a monophyletic group (Fig. 1). The closest genome to the PASGNDM group in the phylogenetic tree is PA_D1 (NZ_CP012585), which belongs to a group of *P. aeruginosa* strains isolated from ventilator-associated pneumonia patients in China^6^.

### Characterizations of PASGNDM345 and PASGNDM699 genomes

**Fig. 2:**
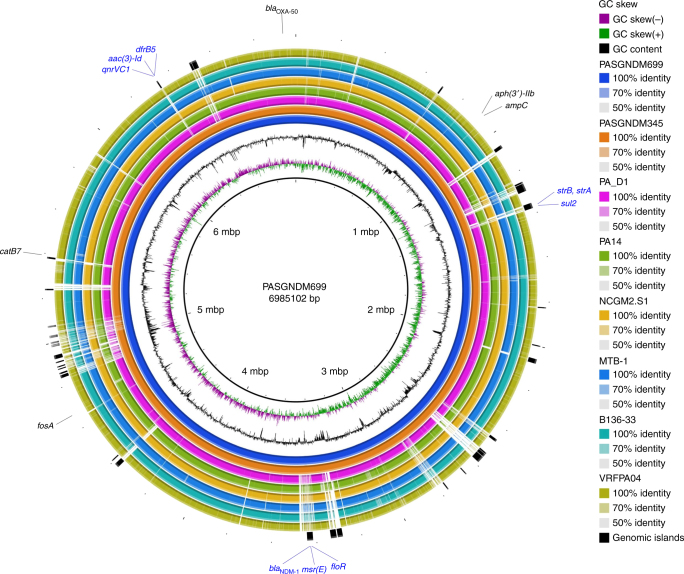
Sequence conservation among PASGNDM699, PASGNDM345 and six other *P. aeruginosa* genomes. From the innermost to outermost: circle 1, PASGNDM699; circle 2, PASGNDM345; circle 3, PA_D1; circle 4, PA14; circle 5, NCGM2.S1; circle 6, MTB-1; circle 7, B136-33; circle 8, VRFPA04. circle 9, Genomic islands present in PASGNDM699 and PASGNDM345. Blocks in black indicate genomic islands present in both PASGNDM699 and PASGNDM345, whereas blocks in gray indicate genomic islands only present in PASGNDM699. Circle 10, predicted antibiotic resistance genes present in PASGNDM699 and PASGNDM345 genomes: black: intrinsic antibiotic resistance genes in *P. aeruginosa* genomes; blue: acquired antibiotic resistance genes. Accession numbers of the genomes are listed in Supplementary Table 2, and the detailed information on the predicted genomic islands is listed in Supplementary Data 1 and 2

The genome of PASGNDM345 consists of a circular chromosome of 6,893,164 bp with an average GC content of 66.1%, whereas the PASGNDM699 genome is 6,985,102 bp with an average GC content of 66.0%. In total, 6503 and 6589 genes were predicted from PASGNDM345 and PASGNDM699 genomes, respectively. It was also noted that PASGNDM699 harbored several genomic islands that are not present in PASGNDM345 or the draft genomes of the other 9 PASGNDM strains (Fig. 2, Supplementary Fig. 1, and Supplementary Data 1).

To identify the strain-specific regions in the PASGNDM genomes, the complete genomes of PASGNDM699 and PASGNDM345 were compared with the six genomes clustered in the same clade in the phylogenetic tree (Fig. 1). Genome alignment result showed that the two PASGNDM genomes possess several regions with low sequence identity to the other six genomes (Fig. 2). In addition, we also predicted the genomic islands in the two PASGNDM genomes by using the Island Viewer 3 server^26^. A total of 41 and 47 genomic islands were predicted from the PASGNDM345 and PASGNDM699 genomes, respectively, which correlate well with the strain-specific regions (Fig. 2, and Supplementary Data 1 and 2). Most of the genes located in the genomic islands encode transposons, efflux pumps and multidrug resistance determinants (Supplementary Data 1 and 2), which may be important for the survival and nosocomial spread of the PASGNDM strains. In addition, all the nine acquired antibiotic resistance genes including *bla*_NDM-1_ are embedded in the genomic islands of PASGNDM699 and PASGNDM345 genomes (Fig. 2), suggesting the importance of mobile genetic elements in the acquisition of antibiotic resistance by the PASGNDM isolates. These acquired antibiotic resistance genes are related to resistance to aminoglycosides (*strA*, *strB* and *aac(3)-Id*), quinolone (*qnrVC1*), sulfoxides (*sul2*), trimethoprim (*dfrB5*), β-lactams (*bla*_NDM-1_), florfenicol (*floR*) and macrolides (*msr(E)*) (Fig. 2). The presence of the acquired antibiotic resistance genes can largely explain the multidrug resistance of the PASGNDM strains (Supplementary Table 1).

### Identification of a novel ICE_Tn*4371*_*6385*

**Fig. 3:**
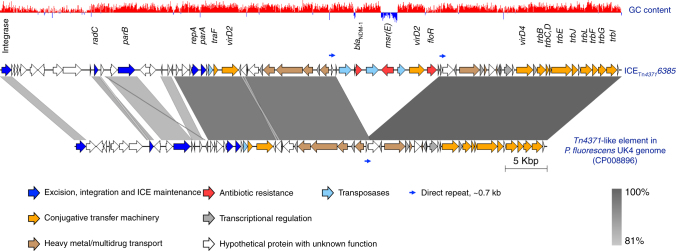
Comparison between ICE_Tn*4371*_*6385* and the Tn*4371*-like element in *P. fluorescens* UK4 genome. The 74 genes encoded by ICE_Tn*4371*_*6385* are shown in arrows with different colors to annotate their functional classes. The length of each arrow is to the scale of the gene size, whereas the arrow direction indicates the transcriptional direction of the gene. The 13.7 kb fragment containing three antibiotic resistance genes are between two direct repeats (indicated by thin blue arrows) in ICE_Tn*4371*_*6385*. The shades between the two elements indicate the sequence identity between the linked regions

It was noted that *bla*_NDM-1_ is clustered together with *msr(E)* and *floR* in the same genomic island (Fig. 2), suggesting that the three antibiotic resistance genes might be co-transferred into the PASGNDM genomes. Further sequence analysis revealed that the three genes are embedded in a 74.2 kb ICE-like element located between the *exoY* (PA2191) and *hcnA* (PA2193) genes in the PASGNDM699 and PASGNDM345 genomes (Fig. 3). Both ends of this element are flanked by a 5′-TTTTTTGT-3′ sequence, which resembles the conserved *attB* site of almost all Tn*4371* family ICEs^25^. It also contains the core genes conserved among the Tn*4371* family ICEs, including an *int* integrase gene, the *parB*, *repA* and *parA* genes of the ICE stabilization system, and homologs to the DNA conjugative transfer machineries such as *traI* and *traG* (Fig. 3)^25^. In addition, the integrase encoded by the *int* gene shared 71% identity with Int_Tn*4371*_ (AJ536756), which further proved that the ICE identified here should be a member of the Tn*4371* family ICEs as suggested by Ryan et al.^25^. We therefore named this element ICE_Tn*4371*_*6385* following the nomenclature system proposed by Roberts et al.^27^.

We searched the ICE_Tn*4371*_*6385* sequence in GenBank and found that it showed similarity to a 56.4 kb ICE-like element present in *Pseudomonas fluorescens* UK4 genome (CP008896). Comparative sequence analysis showed that they share a common Tn*4371* ICE core gene scaffold^25^, whereas their major differences are the unique accessory gene cluster following the *int* gene, and a 13.7 kb fragment carrying the three antibiotic resistance genes in ICE_Tn*4371*_*6385* (Fig. 3). This 13.7 kb fragment is immediately flanked by 695 bp direct repeats, which share 99.87% identity (694/695) (Fig. 3). Interestingly, this direct repeat sequence was also present in the ICE-like element in the *P. fluorescens* UK4 genome (Fig. 3), whereas it showed no similarity to the existing insertion sequence elements as shown by ISfinder^28^. It is possible that the direct repeat sequences in ICE_Tn*4371*_*6385* were the result of homologous recombination between the ancestor of ICE_Tn*4371*_*6385* and a multidrug resistance plasmid, which lead to the acquisition of the 13.7 kb fragment by ICE_Tn*4371*_*6385*.

We therefore present here a novel ICE_Tn*4371*_*6385* element identified in the PASGNDM genomes. ICE_Tn*4371*_*6385* carries three antibiotic resistance genes, which are *bla*_NDM-1_, *msr(E)* and *floR*. To investigate whether *bla*_NDM-1_ is functional in *P. aeruginosa*, we cloned *bla*_NDM-1_ with its promoter sequence into a pME6031 vector to construct a pME6031-NDM plasmid. Transformation of pME6031-NDM into PAO1 and PA14 increases their meropenem minimal inhibitory concentration (MIC) from 1 μg/mL and 0.25 μg/mL to > 64 μg/mL, suggesting that acquisition of *bla*_NDM-1_ by the PASGNDM strains causes their extreme resistance to carbapenems (Supplementary Table 1). Of note, the ICE_Tn*4371*_*6385* sequence is also present in the 9 PASGNDM draft genomes as shown in multiple genome alignment (Supplementary Fig. 1). It was also noted that ICE_Tn*4371*_*6385* has partial homologous sequences in the other genomes (Fig. 2). Further sequence comparison revealed that Tn*4371*-like elements are also present in PA14, MTB-1 and NCGM2.S1 genomes (Supplementary Fig. 2), whereas none of these elements carry any antibiotic resistance genes.

### Msr(E) relieves AZM-mediated quorum sensing inhibition in *P. aeruginosa*

Previous studies reported that sub-MIC concentrations of AZM could suppress the expression of several quorum sensing-regulated virulence factors such as elastase and rhamnolipids and the swarming motility of *P. aeruginosa*, probably by inhibiting its quorum sensing systems^24,29,30^. We therefore hypothesized that acquisition of *msr(E)* by *P. aeruginosa* could counteract the quorum sensing inhibition effect of AZM. However, to investigate the functions of *msr(E)* in the PASGNDM strains by gene deletion is difficult due to their multidrug resistance; hence, an alternative approach by exogenously expressing *msr(E)* in laboratory strains of *P. aeruginosa* was adopted for this purpose. We amplified the *msr(E)* gene together with its putative promoter region from PASGNDM699 genome and inserted the entire fragment into a pUCP18 vector to construct a pUCP18::*msr(E)* plasmid, in which the expression of *msr(E)* is solely controlled by its promoter. Transformation of pUCP18::*msr(E)* into *P. aeruginosa* strains PAO1 and PA14 increased their resistance to AZM by more than 8-fold (from 256 μg/mL to >2048 μg/mL), indicating that *msr(E)* can be expressed from its own promoter and is functional in *P. aeruginosa*.

**Fig. 4:**
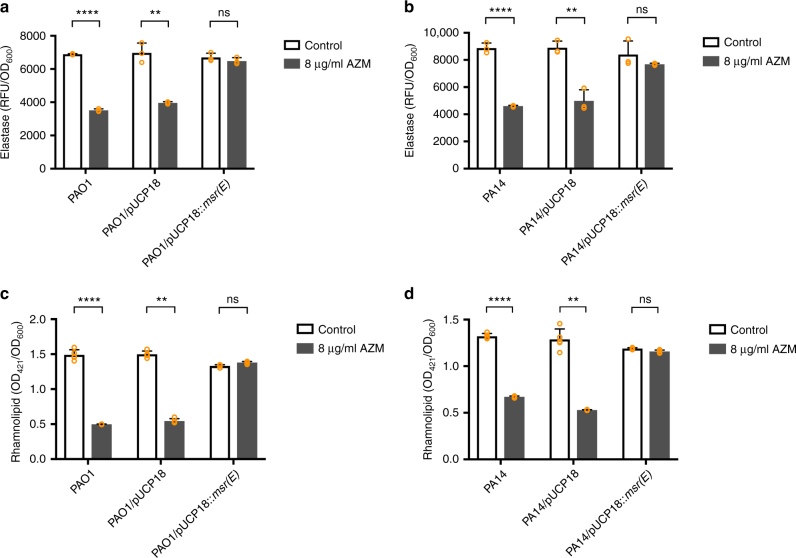
The effect of Msr(E) expression on elastase and rhamnolipid production by PAO1 and PA14 under 8 μg/mL of azithromycin (AZM). Elastase production was inhibited in wildtype and vector-carrying strains of PAO1 (**a**) and PA14 **(b)** under 8 μg/mL AZM, whereas no inhibition was observed in PAO1/pUCP18::*msr(E)* or PA14/pUCP18::*msr(E)* that express Msr(E). Similarly, rhamnolipid production was inhibited in wildtype and vector-carrying strains of PAO1 **(c)** and PA14 (**d**) under 8 μg/mL AZM, whereas no inhibition was observed in PAO1/pUCP18::*msr(E)* or PA14/pUCP18::*msr(E)*. The figure shows relative fluorescence units (RFU) or optical density at 421nm (OD_421_) of the bacterial culture supernatant normalised to optical density at 600 nm (OD_600_) of the bacterial culture. The measurements are taken from distinct samples and mean ± standard deviation is shown in the bar charts, with individual measurement for each sample represented by orange circles. Two-tailed Student’s *t*-test was performed for all the control and treatment groups; *n* = 3 for elastase production assay (**a**, **b**) and *n* = 5 for rhamnolipid quantification assay (**c**, **d**). (**P < 0.01, ****P < 0.0001, ns *P* > 0.05)

**Fig. 5:**
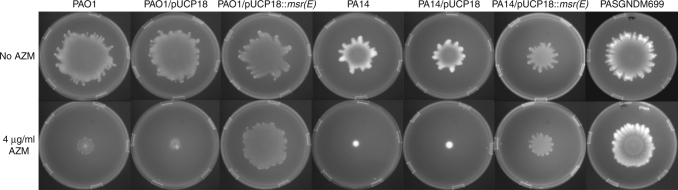
Effect of Msr(E) expression on the swarming motilities of PAO1 and PA14 under 4 μg/mL of azithromycin (AZM). The swarming motility of wildtype and vector-carrying strains of PAO1 and PA14 were inhibited by the addition of 4 μg/mL of AZM, whereas the exogenous expression of Msr(E) in both strains restored the AZM-mediated inhibition on swarming motilities. AZM did not inhibit the swarming motility of PASGNDM699 which harbors *msr(E)*. Figure shows the composite of multiple images

We then performed elastase production, rhamnolipid quantification and swarming motility assays with PAO1/pUCP18::*msr(E)* and PA14*/*pUCP18::*msr(E)* strains. The results showed that 8 μg/mL of AZM (1/32 of the AZM MIC of PAO1) reduced elastase production in both wildtype PAO1 and vector-carrying strain PAO1/pUCP18 by at least 40%, whereas the elastase produced by PAO1/pUCP18::*msr(E)* was not significantly affected upon AZM treatment (Fig. 4a). We also found that 8 μg/mL of AZM reduced rhamnolipid production in both PAO1 and PAO1/pUCP18 by at least 60%, whereas PAO1/pUCP18::*msr(E)* produced similar levels of rhamnolipids in the presence and absence of AZM (Fig. 4c). In addition, the swarming motilities of PAO1 and PAO1/pUCP18 were inhibited by 4 μg/mL of AZM, under which PAO1/pUCP18::*msr(E)* exhibited a normal swarming phenotype (Fig. 5). Similar results were also obtained in the PA14 strains (Figs. 4b, d and 5), suggesting that the resistance to AZM-mediated quorum sensing inhibition by Msr(E) is not strain specific. Taken together, these results clearly showed that the acquisition of *msr(E)* could protect *P. aeruginosa* from AZM-mediated quorum sensing inhibition.

### Msr(E) restores AZM-affected *P. aeruginosa* transcriptome

**Fig. 6:**
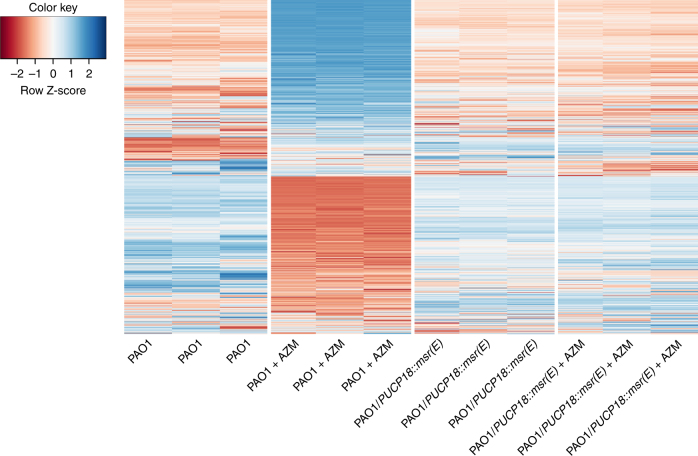
Heatmap of 550 genes that are differentially expressed between the azithromycin (AZM)-treated and non-treated PAO1, and their behaviors in the AZM-treated and non-treated PAO1/pUCP18::*msr(E)*. The differentially expressed genes (fold change >4, *P* value < 0.05) between the AZM-treated and non-treated PAO1 cells were identified by performing a negative binomial test using the DESeq 2 package of R/Bioconductor (*n* = 3). Such changes in the gene expression profiles were not observed between PAO1/pUCP18::*msr(E)-*treated and non-treated with AZM. The complete lists of differentially expressed genes between AZM-treated and non-treated PAO1 and between AZM-treated and non-treated PAO1/pUCP18::*msr(E)* can be found in Supplementary Data 3 and 4

AZM was previously reported to affect the transcriptome of *P. aeruginosa* using microarray analysis^30^. To better show the effect of Msr(E) on the AZM-affected transcriptome of *P. aeruginosa*, we compared the gene expression profiles of PAO1/pUCP18::*msr(E)* in the presence and absence of 8 μg/mL AZM by total RNA sequencing. In PAO1, a total of 550 genes (~10% of PAO1 genome) were differentially expressed upon AZM treatment, of which 305 were upregulated and 245 were downregulated by more than 4-fold (Supplementary Data 3, and Fig. 6). We found that the upregulated genes are enriched in genes encoding ribosomal proteins, the type III secretion pathway apparatus and other defense mechanisms against AZM (*infA* and *efp*), whereas the downregulated genes include *rsmY* and *rsmZ*, the non-coding RNA products of which positively regulate quorum sensing by sequestering the translational inhibitor RsmA, *pslE-I* of the Psl synthesis operon, *lasI* of the LasI/LasR quorum sensing system, and the *flgK* gene the expression of which is essential for swarming motility^31^ (Supplementary Data 3). These results are consistent with findings in previous studies^21,24,29,30,32^ and can explain the quorum sensing inhibition effect of AZM on PAO1 (Figs. 4 and 5). Surprisingly, the transcriptome of PAO1/pUCP18::*msr(E)* was almost not affected by AZM, for which only 6 genes were differentially expressed upon AZM treatment by more than 4-fold (Supplementary Data 4). These results showed that Msr(E) can protect *P. aeruginosa* from AZM-mediated quorum sensing inhibition by restoring the AZM-affected transcriptome.

### Msr(E) abolished anti-Pseudomonas effect of AZM in vivo

**Fig. 7:**
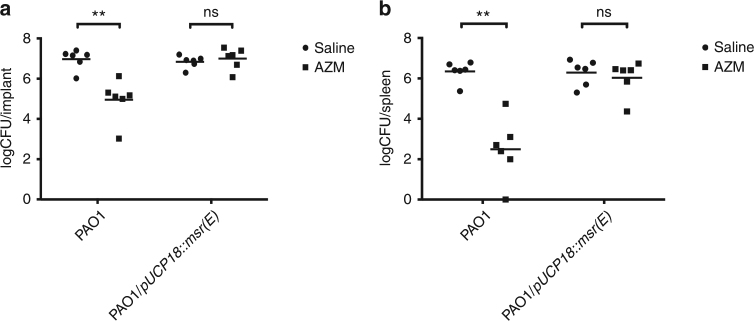
The effect of azithromycin (AZM) treatment on PAO1 and PAO1/pUCP18::*msr(E)* in an in vivo murine infection model. The silicone implants pre-incubated with PAO1 and PAO1/pUCP18::*msr(E)* were inserted into the peritoneal cavity of mice by surgery. AZM treatment at 10 mg per mouse significantly reduced the PAO1 CFU recovered from the silicone implants by 2.0-log (**a**) and the PAO1 CFU recovered from the mouse spleens by 3.8-log (**b**), whereas such reduction was not observed in PAO1/pUCP18*::msr(E)* recovered either from the silicone implants (**a**) or the mice spleens (**b**). Results are shown as log CFU per silicone implant and log CFU per mouse spleen, respectively (*n* = 6; **P < 0.01; ns *P* > 0.05; Mann–Whitney test, two-sided)

Macrolide antibiotics such as erythromycin and clarithromycin were previously shown to enhance the clearance of *P. aeruginosa* at the infection sites in a murine implant infection model and a murine lung infection model^33,34^. To investigate if the expression of *msr(E)* in *P. aeruginosa* can affect the anti-Pseudomonas activity of AZM in vivo, we used the murine implant model to compare the effect of AZM treatment on PAO1 and PAO1/pUCP18::*msr(E)* infections. Briefly, silicone implants pre-colonized with PAO1 or PAO1/pUCP18::*msr(E)* were inserted into the peritoneal cavity of mouse. After 12 h of incubation, mice were treated with AZM by injecting AZM solution into the peritoneal cavity, whereas the control mice were injected with the same amount of saline. The silicone implants and mice spleens were harvested at 24 h post infection to enumerate colony-forming units (CFUs) of *P. aeruginosa*, which were used to indicate the clearance of bacteria at infection site and the spreading of infection to other organs according to previous studies^34,35^. We found that AZM treatment could reduce the CFUs of PAO1 residing on the silicone implants and in the mice spleens by 2.0-log and 3.8-log, respectively (Fig. 7). By contrast, AZM treatment did not significantly affect the CFU of PAO1/pUCP18::*msr(E)* recovered from either the silicone implants or the mice spleens (Fig. 7). These results indicated that Msr(E) could confer resistance against the anti-Pseudomonas activity of AZM in vivo on *P. aeruginosa*.

## Discussion

Tn*4371* family ICEs have been previously described in a broad range of β- and γ-proteobacteria isolated from both environmental and clinical settings and confer their host adaptive functions such as multidrug and heavy metal resistance, as well as the ability to metabolize specific carbon sources^25^. In addition, a Tn*4371*-like element from *P. aeruginosa* genomes has recently been characterized to carry *bla*_SPM-1_ and *bcr1*, suggesting that the Tn*4371* family ICE is an important source of carbapenem resistance in *P. aeruginosa*^36^. In the current work, we identified a novel ICE_Tn*4371*_*6385* from PASGNDM genomes isolated from clinical sources. This element carries three antibiotic resistance genes including *bla*_NDM-1_, *msr(E)* and *floR*, and retains the machinery for conjugative transfer (Fig. 3), suggesting that it is responsible for the transmission of these resistance genes into the PASGNDM genomes^25,37^. However, the conjugative transfer of ICE_Tn*4371*_*6385* was not observed in mating experiments between PASGNDM699 and PAO1 or PA14 strains on agar plates (Methods). It is possible that the conjugation has extremely low efficiencies that is below our detection limit or requires specific inductive conditions^37,38^. To the best of our knowledge, this is the first description of *bla*_NDM-1_ to be carried by ICEs.

The macrolide resistance gene *msr(E)* were mainly identified from Gram-negative bacteria such as Enterobacteriaceae spp., *Pasteurella multocida* and *A. baumannii*^39–41^, but has not been described in *P. aeruginosa* before. Hence, the transmission of *msr(E)* by ICE_Tn*4371*_*6385* into the PASGNDM genomes led us to investigate its functional roles in *P. aeruginosa*. Our in vitro assays clearly showed that the exogenous expression of *msr(E)* in *P. aeruginosa* abolishes the quorum sensing inhibition activity of AZM in vitro, which in turn rescued the inhibition of elastase production, rhamnolipid production and swarming motility by sub-MIC levels of AZM (Figs. 4 and 5). In addition, the acquisition of *msr(E)* by PAO1 almost completely restored the AZM-affected transcriptome and abolished the anti-Pseudomonas activity of AZM in a murine infection model (Figs. 6 and 7, Supplementary Data 3 and 4). These results demonstrate that Msr(E) could confer *P. aeruginosa* with resistance to AZM-mediated quorum sensing inhibition, which is an important treatment strategy for *P. aeruginosa* infections^42^. The transmission of *msr(E)* into this organism will greatly challenge the use of AZM in treating infections caused by *P. aeruginosa*.

While we showed that Msr(E) can abolish the quorum sensing inhibition effect of AZM, the mechanism behind remains elusive. The 491-amino acid protein Msr(E) is a member of the ABC-F subfamily of adenosine triphosphate (ATP)-binding cassette proteins, which are characterized by two ATP-binding cassette domains connected by an ~80 amino acid linker sequence^43,44^. It was shown that members of the ABC-F proteins can displace antibiotics from ribosomes in vitro, which rescued translation from antibiotic-mediated inhibition^44^. In addition, ribosomal protection was shown to be sufficient to prevent AZM-mediated quorum sensing inhibition in *P. aeruginosa* in a previous study^45^. We therefore propose that Msr(E) can inhibit the binding of AZM with ribosomes, thereby indirectly restoring AZM-mediated quorum sensing inhibition. We are currently investigating on the mechanisms of Msr(E)-mediated macrolide resistance through structural biology approaches.

The third antibiotic resistance gene carried by ICE_Tn*4371*_*6385* is *floR*, which encodes an efflux pump and is the determinant for florfenicol resistance^46^. This gene was first identified in *Salmonella typhimurium* DT104 in 1999 and has ever since been detected in *Escherichia coli and P. multocida* isolated from livestock and aquaculture settings^47–50^. It is possible that the emergence and spreading of *floR* is due to the selection by florfenicol, since this antibiotic has been extensively used in veterinary medicine, especially in aquaculture settings^49,51^. Therefore, the carriage of *floR* by ICE_Tn*4371*_*6385* may associate with survival advantages under environmental conditions where florfenicol may be present^51,52^, and hence facilitate the transmission and dissemination of *bla*_NDM-1_ and *msr(E)*.

In conclusion, we characterized the full-length genomes of two NDM-1-producing *P. aeruginosa* clinical isolates, from which we identified a novel ICE_Tn*4371*_*6385* element encoding three antibiotic resistance genes. Among them, the *bla*_NDM-1_ gene is responsible for extreme resistance to carbapenems, whereas *msr(E)* can restore AZM-mediated quorum sensing inhibition, and *floR* may enhance the survival of host bacteria under florfenicol exposure. To our knowledge, we present the first description of *bla*_NDM-1_ to be carried by ICEs, and the first evidence that Msr(E) confers resistance to macrolide-mediated quorum sensing inhibition on *P. aeruginosa*. Our findings highlight the importance of ICEs in transmitting antibiotic resistance, and that anti-virulence treatment of *P. aeruginosa* infections by targeting quorum sensing through sub-inhibitory concentrations of macrolide antibiotics can be challenged by horizontal gene transfer.

## Methods

### Bacterial strains and plasmids

All the strains were routinely grown in Lysogeny broth (LB) or on LB plates with 1.5% agar at 37 °C. The *msr(E)* gene with its putative promoter were amplified from PASGNDM699 genome using primers 5′-ACCGGCCAAGATAGTTGACG-3′ and 5′-AGGAAGTTCAACCGCCCTTT-3′ and ligated into the *sma*I site of pUCP18^53^ to construct a pUCP18*::msr(E)* plasmid, in which *msr(E)* is upstream to the *lac* promoter of pUCP18. Similarly, *bla*_NDM-1_ and the 120 bp upstream sequence containing the promoter^54^ was amplified using primers 5'-ATCTATCGATGCATGCCATGCTGTCGCACCTCATGTTT-3' and 5'- ATGACGTCGACGCGTCTGCAAGCCCAGCTTCGCATAA-3'. The PCR product was ligated in between *Nco*I and *Pst*I sites of a pME6031 vector^55^ to construct a pME6031-NDM plasmid, in which the expression of *bla*_NDM-1_ is solely controlled by its own promoter. Plasmids were transformed into PAO1 and PA14 by electroporation, and the transformants were selected and grown in LB supplemented with appropriate antibiotics.

### Sequencing and annotation

Genomic DNA was purified using Blood and Cell Culture DNA Midi Kit (Qiagen) and sequenced on an Illumina MiSeq platform or a PacBio RS II system. The full-length genomes of PASGNDM345 and PASGNDM699 were assembled from long reads obtained from the PacBio RS II system using HGAP2 pipeline assisted with manual curation to resolve repetitive region in the assembly. The ICE_Tn*4371*_*6385* sequence and ICE-like element from *P. fluorescens* UK4 were uploaded to the Rapid Annotations using Subsystem Technology (RAST) server^56^ for gene prediction and annotation, assisted with manual BLASTp search. Comparison of the two sequences was done by BLASTn search using EasyFig v2.2.2^57^.

### Phylogenetic and comparative genomic analysis

Core-genome alignment of the *P. aeruginosa* genomes was performed using Parsnp v1.1.2^58^. The phylogenetic inference was carried out on the variant sites using approximate maximum likelihood algorithm by using FastTree 2 integrated in Parsnp^59^, with clade confidence estimated with SH-like supporting values. The branch length in the phylogenetic tree represent substitutions per core-genome site. Genomic islands of PASGNDM699 and PASGNDM345 were predicted by the IslandViewer 3 server^60^, and antibiotic resistance genes were predicted using the ResFinder 2.1 server^61^. Comparison of PASGNDM699 and PASGNDM345 genomes with six other full-length *P. aeruginosa* genomes was done by BLASTn search using BLAST Ring Image Generator v0.95^62^, with genomic islands and antibiotic resistance genes labeled in their respective locations. Multiple sequence alignment analysis was performed by using ProgressiveMauve integrated in Mauve v2.4^63^.

### Elastase and rhamnolipid quantification

Bacterial strains were grown in ABT minimal medium^64^ supplemented with 5 g/L glucose and 2 g/L casamino acids (ABTGC), with or without addition of 8 μg/mL AZM (Spectrum). The supernatants of overnight culture were filter sterilized before quantification of elastase and rhamnolipid production. Elastase activity was measured using EnzCheck Elastase Assay Kit (Thermo Fisher Scientific) according to the manufacturer’s instructions. The quantification of rhamnolipid was performed as previously described^65^ with modifications. Briefly, rhamnolipid was extracted from 1 mL of filtered supernatants with 2 mL of diethyl ether. Then, 1 mL of the diethyl ether extract was collected, vacuum dried and re-dissolved in 50 μL of deionized water. The solution was added with 450 μL of 0.19% (w/v) orcinol dissolved in 53% H_2_SO_4_, followed by heating at 80 °C for 30 min. The elastase activity and rhamnolipid production were quantified by measuring emission at 530 nm upon excitation at 485 nm and absorbance at 421 nm by using an Infinite 200 PRO system (Tecan), respectively. The results were normalized with optical density at 600 nm (OD_600_) of the bacterial culture. Both elastase and rhamnolipid quantification assays were successfully replicated for at least three times, with measurements from distinct samples shown in the figure.

### Swarming motility assay

The swarming motility of each strain was measured on 0.5% agar plates containing 8 g/L nutrient broth (Oxoid) and 5 g/L glucose^66^. Then, 1 μL of bacterial overnight culture (adjusted to OD_600_ = 1) was inoculated onto the center of the plate, followed by incubation at 37 °C for 12 h. The images were taken using a Gel Doc XR+ System (Bio-Rad). This experiment was successfully replicated for three times.

### Transcriptomic analysis

Strains were grown in ABTGC medium at 37 °C with 200 rpm shaking. Cells were harvested at mid-log phase (OD_600_ = 0.6), and the total RNA was extracted using RNeasy Mini Kit (Qiagen). RNA sequencing was performed on an Illumina HiSeq 2500 platform to generate paired-end reads of 100 nt, which were trimmed and mapped to PAO1 genome (NC_002516.2) by using CLC Genomics Workbench 9.0 (Qiagen). The transcript count table was analyzed by DESeq 2 package of the R/Bioconductor by performing negative binomial test^67^. Hierarchical clustering analysis was performed to produce the heatmap for the differentially expressed genes with statistical significance (fold change>4, *P* < 0.05) using heatmap.2 package of the R/Bioconductor^68^.

### Animal model

A murine silicone implant model was used to evaluate effects of in vivo AZM treatment on the two strains as previously described with modifications^35^. Briefly, bacterial cells were re-suspended in 0.9% NaCl to an OD_600_ of 0.01. Bacteria cells were allowed to attach onto sterilized silicone tubes (length, 3 mm; inner diameter, 4 mm; outer diameter, 6 mm; Ole Dich) by incubation at 37 °C, with 110 rpm shaking for 18 h, after which the silicone tubes coated with bacteria were implanted into mouse peritoneal cavity by surgery. Treatment with AZM (10 mg per mouse, dissolved in 0.2 mL of 0.9% saline) or saline was performed by injection into the mouse peritoneal cavity 12 h after surgery. Mice were killed 24 h after surgery. Bacterial cells residing on the silicone tube and in the mouse spleen were suspended into 0.9% NaCl solution by sonication using an Elmasonic P120H (Elma, Germany; power = 50% and frequency = 37 KHz) and homogenization using a Bio-Gen PRO200 Homogenizer (Pro Scientific), respectively. CFU was quantified by serial dilution and plating on LB agar plates, and the results were shown in log CFU.

### Mating experiment

The mating experiment between PASGNDM699 and both PAO1 and PA14 was performed to investigate if ICE_Tn*4371*_*6385* can transfer into other *P*. *aeruginosa* strains by conjugation. The recipient strains PAO1 and PA14 were transformed with pME6031 vector by electroporation to confer tetracycline resistance for selection. The donor and recipient strains were mixed in a 1:1 ratio and spotted onto a sterilized filter paper placed on agar plates. Conjugation experiments were performed on both rich medium (LB) and minimal medium (ABTG), and at both 25 °C and 37 °C. Transconjugants were selected on LB agar plates containing tetracycline (60 μg/mL) and meropenem (5 μg/mL).

### Ethics

The use of clinical specimen samples was approved by Department of Laboratory Medicine, National University Hospital, Singapore, registered under the reference 2016/00856. The animal model protocols were approved by the Institutional Animal Care and Use Committee of the Nanyang Technological University, under the permit number A-0191 AZ. Animal experiments were performed in accordance to the NACLAR (National Advisory Committee for Laboratory Animal Research) Guidelines of Animal and Birds (Care and Use of Animals for Scientific Purposes) Rules by Agri-Food & Authority of Singapore (AVA).

### Statistics

All experiments were performed in at least three biologically independent replicates, with numbers of replicates indicated with “*n*” in figure legends. The statistical significance test method used for each experiment is also indicated in respective figure legend.

### Data availability

The draft and complete genome sequences have been deposited in GenBank (www.ncbi.nlm.nih.gov/genbank/) under BioProject PRJNA381838. The complete genomes of PASGNDM699 and PASGNDM345 are CP020704 and CP020703, respectively. The genome sequencing and RNA-Seq raw reads are deposited in the NCBI Short Read Archive database (www.ncbi.nlm.nih.gov/sra) with the accession codes SRP103165 and SRP103155, respectively. All other relevant data are available from the authors upon reasonable request.

## Electronic supplementary material

Supplementary Information

Description of Additional Supplementary Files

Supplementary Data 1

Supplementary Data 2

Supplementary Data 3

Supplementary Data 4
