# Supplementary material for: Acquisition of resistance to carbapenem and macrolide-mediated quorum sensing inhibition by Pseudomonas aeruginosa via ICETn43716385
Source: Commun Biol. 2018 May 31;1:57. doi: 10.1038/s42003-018-0064-0 (PMC6123621; doi:10.1038/s42003-018-0064-0)
Supplement: Supplementary file 1 — Supplementary Information [file 42003_2018_64_MOESM1_ESM.pdf]

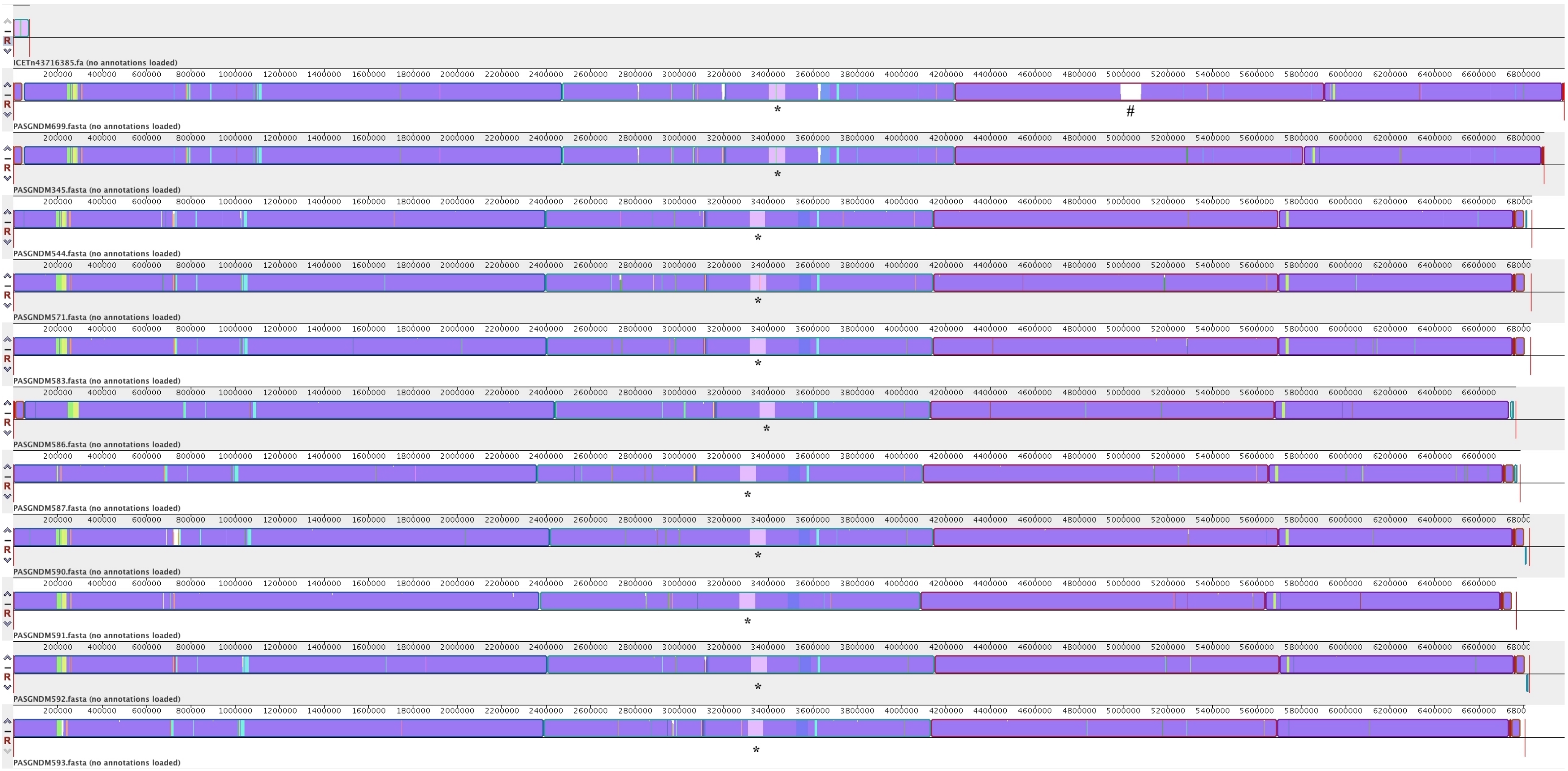

Supplementary Figure 1. Alignment of the 11 PASGNDM genomes and ICE$_{Tn4371}$*6385*. Complete genomes of PASGNDM345 and PASGNDM699 were aligned with draft genomes of the 9 PASGNDM strains using Progressive Mauve as described in the methods. The ICE$_{Tn4371}$*6385* sequence was also included in the alignment (the first row), and the homologous regions of ICE$_{Tn4371}$*6385* were found in the draft genomes of the 9 PASGNDM strains (indicated by *). Strain specific region in PASGNDM699 but not the other PASGNDM genomes are indicated by #, which can be explained by the acquisition of several novel genomic islands (Supplementary Data 1).

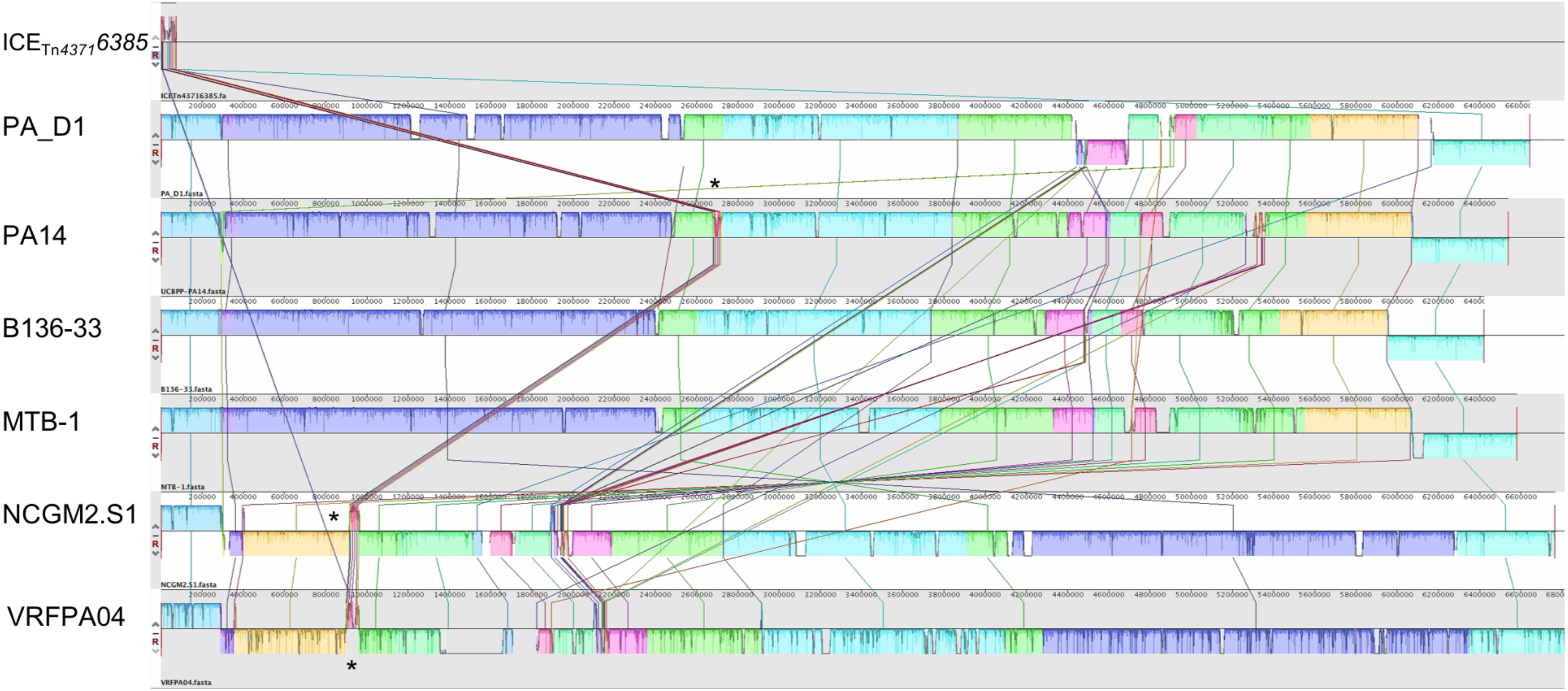

Supplementary Figure 2. Alignment of ICE$_{Tn\mathit{4371}}$*6385* and the other six genomes used for their comparison in Figure 2. The sequence of ICE$_{Tn\mathit{4371}}$*6385* was aligned with the six genomes to find its homologous sequences using Progressive Mauve as described in the methods. Homologous regions in different genomes are indicated by the same colour and connected by lines. BLASTn search against the ICEberg v1.0 database (http://db-mml.sjtu.edu.cn/ICEberg/) suggested that the homologous sequences present in PA14, NCGM2.S2 and VRFPA04 genomes (indicated by *) are Tn*4371*-like elements, which are 40.9 kb, 47 kb and 61 kb, respectively. Positions of the Tn*4371*-like elements in their respective genomes are: PA14 (2678237 – 2718843 nt), NCGM2.S2 (912944 – 959934 nt), and VRFP04 (895679 – 956428 nt).

Supplementary Table 1. Minimal inhibitory concentrations (µg/ml) of PASGNDM strains to various antibiotics. These strains only remain sensitive to polymyxin B. Clinical specimens from which the strains were isolated are also shown in the table.

Strain	Specimen	Ceftazidime	Cefepime	Imipenem	Meropenem	Gentamicin	Amikacin	Ciprofloxicin	Levofloxacin	Ertapenem	Polymyxin B	Azithromycin
PASGNDM544	Endotracheal aspirate	>64	>64	>16	>16	>16	>64	>4	>8	>8	1.5	>512
PASGNDM699	Sputum	>64	>64	>16	>16	>16	>64	>4	>8	>8	1.5	>512
PASGNDM345	Sputum	>64	>64	>16	>16	>16	>64	>4	>8	>8	2	>512
PASGNDM571	Urine	>64	>64	>16	>16	>16	>64	>4	>8	>8	1	>512
PASGNDM583	Urine	>64	>64	>16	>16	>16	>64	>4	>8	>8	1	>512
PASGNDM586	Urine	>64	>64	>16	>16	>16	>64	>4	>8	>8	0.5	>512
PASGNDM587	Wound swab	>64	>64	>16	>16	>16	>64	>4	>8	>8	0.5	>512
PASGNDM590	Urine	>64	>64	>16	>16	>16	>64	>4	>8	>8	0.75	>512
PASGNDM591	Urine	>64	>64	>16	>16	>16	>64	>4	>8	>8	2	>512
PASGNDM592	Urine	>64	>64	>16	>16	>16	>64	>4	>8	>8	2	>512
PASGNDM593	Urine	>64	>64	>16	>16	>16	>64	>4	>8	>8	0.75	>512

Supplementary Table 2. Genomes and their accession numbers used to construct the phylogenetic tree shown in Figure 1.

Strain name	GenBank Accession number
DK2	NC_018080.1
PA14	NC_008463.1
PAO1	NC_002516.2
B136-33	NC_020912.1
LESB58	NC_011770.1
M18	NC_017548.1
NCGM2.S1	NC_017549.1
RP73	NC_021577.1
YL84	NZ_CP007147.1
MTB-1	NC_023019.1
Carb01-63	NZ_CP011317.1
VRFPA04	NZ_CP008739.1
PA_D1	NZ_CP012585.1
DHS01	NZ_CP013993.1
SJTD-1	NZ_CP015877.1
AES-1R	NZ_CP013680.1
ATCC 27853	NZ_CP015117.1
USDA-ARS-USMARC-41639	NZ_CP013989.1
SCV20265	NC_023149.1
PA1	NC_022808.2
NCTC10332	NZ_LN831024.1
