# Supplementary material for: Acquisition of resistance to carbapenem and macrolide-mediated quorum sensing inhibition by Pseudomonas aeruginosa via ICETn43716385
Source: Commun Biol. 2018 May 31;1:57. doi: 10.1038/s42003-018-0064-0 (PMC6123621; doi:10.1038/s42003-018-0064-0)
Supplement: Supplementary file 2 — Description of Additional Supplementary Files [file 42003_2018_64_MOESM2_ESM.docx]

**Description of Additional Supplementary Files**

File Name: Supplementary Data 1

Description: Predicted genomic islands in PASGNDM699 genome.

File Name: Supplementary Data 2

Description: Predicted genomic islands in PASGNDM345 genome.

File Name: Supplementary Data 3

Description: The 550 differentially expressed genes between azithromycin-treated and non-treated PAO1.

File Name: Supplementary Data 4

Description: Six differentially expressed genes between azithromycin-treated and non-treated PAO1/pUCP18*::msr(E)*.
